# Considerations for the clinical development of immuno-oncology agents in cancer

**DOI:** 10.3389/fimmu.2023.1229575

**Published:** 2023-08-11

**Authors:** Atanasio Pandiella, Emiliano Calvo, Victor Moreno, Eitan Amir, Arnoud Templeton, Alberto Ocana

**Affiliations:** ^1^ Centro de Investigación del Cáncer, CIC-CSIC, Salamanca, Spain; ^2^ Centro de Investigación Biomédica en Red en Oncología (CIBERONC), Madrid, Spain; ^3^ START Madrid-HM Centro Integral Oncológico Clara Campal (CIOCC), Early Phase Program, HM Sanchinarro University Hospital, Madrid, Spain; ^4^ START Madrid-Fundación Jiménez Díaz (FJD) Early Phase Program, Fundación Jiménez Díaz Hospital, Madrid, Spain; ^5^ Division of Medical Oncology & Hematology, Department of Medicine, Princess Margaret Cancer Centre and University of Toronto, Toronto, ON, Canada; ^6^ Department of Medical Oncology, St. Claraspital, Basel, Switzerland; ^7^ Experimental Therapeutics Unit, Medical Oncology Department, Hospital Clínico San Carlos (HCSC), Instituto de Investigación Sanitaria (IdISSC), Madrid, Spain

**Keywords:** immunotherapy, drug development, PDL1, CTLA4, LAG3, T-cell engagers, cancer

## Abstract

Targeting of the immune system has shown to be a successful therapeutic approach in cancer, with the development of check point inhibitors (ICI) or T-cell engagers (TCE). As immuno-oncology agents modulate the immune system to attack cancer cells and do not act directly on oncogenic vulnerabilities, specific characteristics of these compounds should be taken in consideration during clinical development. In this review we will discuss relevant concepts including limitations of preclinical models, special pharmacologic boundaries, clinical development strategies such as the selection of clinical indication, line of treatment and backbone partner, as well as the endpoints and expected magnitude of benefit required at different stages of the drug development. In addition, future directions for early and late trial designs will be reviewed. Examples from approved drugs or those currently in clinical development will be discussed and options to overcome these limitations will be provided.

## Introduction

Immunotherapy has a central role in the treatment of cancer with the approval of several immuno-oncology (IO) agents in different indications. Trials supporting the approval of these drugs has demonstrated that acting on the immune system can be a successful therapeutic approach (1). Beyond the use of cell therapy like CAR-T cells, several strategies, mainly using antibodies or antibody formats, have demonstrated clinical activity. Anti PD-(L)1, anti CTLA4 and anti LAG3 antibodies typically termed as immune checkpoint inhibitors (ICI), and Bi-specific T-cell engagers (TCE) have shown clinical activity in different indications, and it is anticipated that over the next few years several other agents with similar mechanism of action will demonstrate efficacy (2, 3). However, the clinical development of these compounds differs from small molecules or chemotherapies. These agents do not act directly on tumor cells, but on cellular components of the host. In addition, activation of the immune system has a characteristic efficacy and safety profile with both acute and long-term side effects (4). Given the fact that when acting on one target there is a modulation of other cellular populations (5, 6), and in many occasions, these targets are shared between different cell types, the ability to identify and develop biomarkers in immune-oncology is more challenging than for agents targeting oncogenic vulnerabilities (7, 8). Finally, for some patients and indications, given the extraordinary activity observed with some compounds, an accelerated approval has been granted, speeding patient access to these therapies, but also requiring confirmatory registration phase III studies. This adds uncertainty about the real clinical value of the agent when explored in early stage studies (9).

In this article, we describe the current status, limitations and options for improvement for the clinical development of immunotherapy in cancer including: (i) the limitations of preclinical models to predict biological activity in humans (ii) special pharmacologic considerations for the development of these agents (iii) the selection of indication, line of treatment and backbone partner, and (iv) the threshold of activity that has to be reached for the compound to be considered as clinically meaningful (10).

## Lack of preclinical models to predict human clinical activity

When evaluating therapeutic compounds against oncogenic vulnerabilities or cytotoxic chemotherapy, the efficacy of these agents requires evaluation using *in vivo* models (1, 11). In this case, several models can be used, including nude mice with xenografted tumor cells, transgenic mice with a specific genomic alteration, or patient derived xenograft (PDX) models. Generally, it is considered that the effect observed in these models can mirror the potential activity detected in humans (1, 12). In contrast, for immunotherapy agents, it is generally accepted that preclinical *in vivo* data do not translate into clinical efficacy in patients (10, 13). The use of syngeneic mice models where the animal immune system is preserved has been utilized extensively, and we have seen this model incorporated in the evaluation of agents approved recently (14). A detailed review of models that recreate the human immune system is beyond the scope of this review and can be found in other articles (10). In this context, although very sophisticated models have been developed with the intent to reflect the human immune system, it is generally accepted that none of these models can predict the efficacy of the evaluated compound when tested in humans (10). Similarly, *in vivo* models do not predict safety for later human studies, therefore the US Food and Drug Administration (FDA) has decided not to make animal studies mandatory for investigational new drug (IND) applications of novel agents (15). This initiative, which was released recently, endorses the limited information of some of these pre-clinical models, including those to evaluate IO agents. As a consequence, models for testing efficacy *in vitro* like the use of tumor organoids or tissue cross reactivity studies for safety (among others), are gaining interest (16).

## Pharmacological properties and safety of IO agents

Several concepts must be taken in consideration when developing novel IO agents in cancer. For instance, from a pharmacokinetic (PK) perspective, if the target is significantly expressed in non-transformed tissue or is abundant in immune cells not located in tumor areas, a phenomenon called target mediated drug disposition (TMDD) can be observed. This translates to a reduction of the exposure of the compound as the agent binds first to targets not expressed within tumor areas (17). This effect has also been termed “sink effect” on account of the reduction of the compound in plasma. To avoid this phenomenon, more frequent administrations of the agent are needed during the first cycles to saturate target binding in non-tumor areas (17). TMDD is observed frequently with many IO agents including most of the CD3 T-cell engagers, CD73 inhibitors or 4-1BB bi-specific antibodies, among others (18).

An additional problem is the development and presence of anti-drug antibodies (ADA) against biologic or protein-based drugs. Although there are several non-clinical pharmacology methods to predict the development of ADA in humans, it is impossible to accurately predict the potential impact that ADAs will have in patients by neutralizing the new compound (19). Overall, complex protein structures that do not mimic human formats have higher chances for the development of ADAs (20). Recent examples have demonstrated how the production of ADAs can limit the development of novel agents particularly when their presence modifies the PK exposure and therefore impacts target engagement (21, 22). In this case, only the administration of doses that can saturate the capacity to produce ADAs can overcome this limitation. This requires administration of the agent at higher doses, but this can only be achieved if there is a sufficient therapeutic index, a condition not observed with all agents (20). Of note, agents that activate CD4+ T-cells and therefore support humoral response can have a higher probability to induce ADA (23, 24) **Table 1** describes elements that can influence PK and therefore affect target engagement.

**Table 1:** Elements that can influence pharmacokinetics and target engagement.

Elements	Explanation	Situation	Mitigation	Examples
TMDD Target mediated drug dispositioning	The investigational agent binds first to the target expressed in non-tumor areas	More frequently observed when a target is expressed in non-tumor areas	More frequent administrations of the compound to saturate the receptor	Most T cell engagers, bi-specifics like PD1-41BB, etc
ADAs Anti-drug antibodies	Antibodies produced by the own immune system against the investigational agent that neutralize their activity	More frequently observed with compounds not following a physiological protein structure	Increase dose levels to saturate ADAs. Difficult to perform if there is a narrow therapeutic index	Complex protein structures, more frequent with bi-specifics.
Modulation of the expression of the target with the compound	When one target can modulate immune populations that expressed the other target	More frequently observed with bi-specifics, acting on two different targets expressed in different populations.	Identify the correct biological active dose through the use of PK/PD modelling	Particularly bi-specifics like PDL1-OX40 agents, among others

Finally, management of side effects is particularly important for T-cell activators/engagers, where presence of cytokine release syndrome (CRS), neurologic toxicity or infusion reactions (IR) can limit their development (25). With this regard, premedication with steroids, treatment with anti-IL-6 inhibitors, step up schedule approaches, subcutaneous administrations or the pre-administration with anti-CD20 antibodies, have been implemented in an intent to reduce toxicity and facilitate the development of these agents (5, 26). **Table 2** describes strategies to optimize and reach optimal biological active doses overcoming the main limitation of toxicity.

**Table 2:** Strategies to optimize the clinical development of TCE.

Strategy	Rationale	Action	Effect	Examples
Step-up dosing	Priming the immune system in a more gradual manner	Incremental increasing of the dose before reaching the target dose level	Reduce toxicities including CRS and neurotoxicity	Most of the current approved TCE including epcoritamab and teclistamab
Subcutaneous administration	Slower and lower peak drug concentrations (Cmax)	Reduce activation of the immune system due to a gradual release of the drug in the circulation	Reduce toxicities including CRS and neurotoxicity	Most of the TCE in clinical development at this moment
Modifications in the molecular structure	Lower affinity to CD3 Increase the valency to TAA arm	Induce less activation of T cells and distribution between tumor and Lymphoid tissue	Reduce toxicities including CRS and neurotoxicity	Ongoing studies, example REGN5458, REGN5459
PK/PD modulation	Clinical activity does not relate with receptor occupancy (RO)	PK/PD modulation considering bell shape effect. Quantitative system pharmacology (QSP) (27, 28).	Identify the optimal RP2D	Exposure-response analysis of glofitamab demonstrated clinical activity with only 1% RO of CD20 (29)

## Selection of indication, line of treatment, combo partner and early trial design

Only two types of IO compounds have been approved for the treatment of hematologic malignancies and solid tumors; and those include TCE and ICI. TCE are bi-specific antibodies that link CD3 or any other T-cell functional receptor with a tumor associated antigen (TAA) to induce tumor cell death by the effector immune cell (30). Here, a differential expression of TAA is mandatory to avoid non-tumor, on-target toxicity. Current approved TCE are designed against well-defined TAA in selected indications, for instance CD3-CD19 bi-specifics including blinatumomab in Philadelphia chromosome-negative relapsed or refractory precursor B-cell acute lymphoblastic leukemia (R/R ALL) (31), and adults and children with B-cell precursor acute lymphoblastic leukemia (BCP ALL) in first or second complete remission with minimal residual disease (MRD) (32), or more recently teclistamab, in relapse or refractory Multiple Myeloma for B-cell maturation antigen (BCMA)-CD3 (33, 34). CD3-CD20 mosunetuzumab has received accelerated approval for the treatment of relapsed or refractory follicular lymphoma after two or more lines of treatment (35, 36). Epcoritamab, a bispecific antibody targeting CD3 and CD20, has received FDA priority review for the treatment of relapsed/refractory diffuse large B cell Lymphoma (37). The development of TCE can be more successful in hematologic malignancies where monoclonal expansion of tumor cells drives the disease, and TAA are homogeneously expressed (e.g. CD19 or CD20 in B cell lymphoma) (38). Identification of specific TAA in solid tumors is more challenging, due to intra- and inter-tumor heterogeneity (39). However, interestingly some TAA in solid tumors, are specifically expressed like KLK2 in prostate cancer or LY6G6D in colorectal cancer. These are therefore promising candidates for the development of TCE (40–42). Regarding the line of treatment and backbone partner, given that these compounds induce a profound T-cell activation with significant immunologic toxicity, later lines of treatment are chosen for evaluation, and usually are administered in monotherapy, and only evaluated in combination once the optimal dose, schedule and route of administration is clearly defined (5, 43).

As described before, ICI such as anti PD(L)1, anti CTLA4 have been part of the therapeutic armamentarium for over a decade. More recently the anti-LAG3 antibody relatlimab was approved in first line melanoma (44). Most of these agents have demonstrated activity in late lines of treatment particularly in patients with immune reactive tumors (2). Once these agents show activity in patients pretreated after several lines of standard treatments, evaluation of efficacy in earlier lines, either alone or in combination with standard of care agents is warranted. These include combinations with chemotherapy regimens in first-line gastric, esophageal, non-small cell lung cancer (NSCLC) or triple negative breast, among other tumors. Additionally, examination as monotherapy in PD-L1 enriched populations like in Head and Neck Squamous Cell Carcinoma (HNSCC) or NSCLC is also of interest (9).

**Figure 1:**
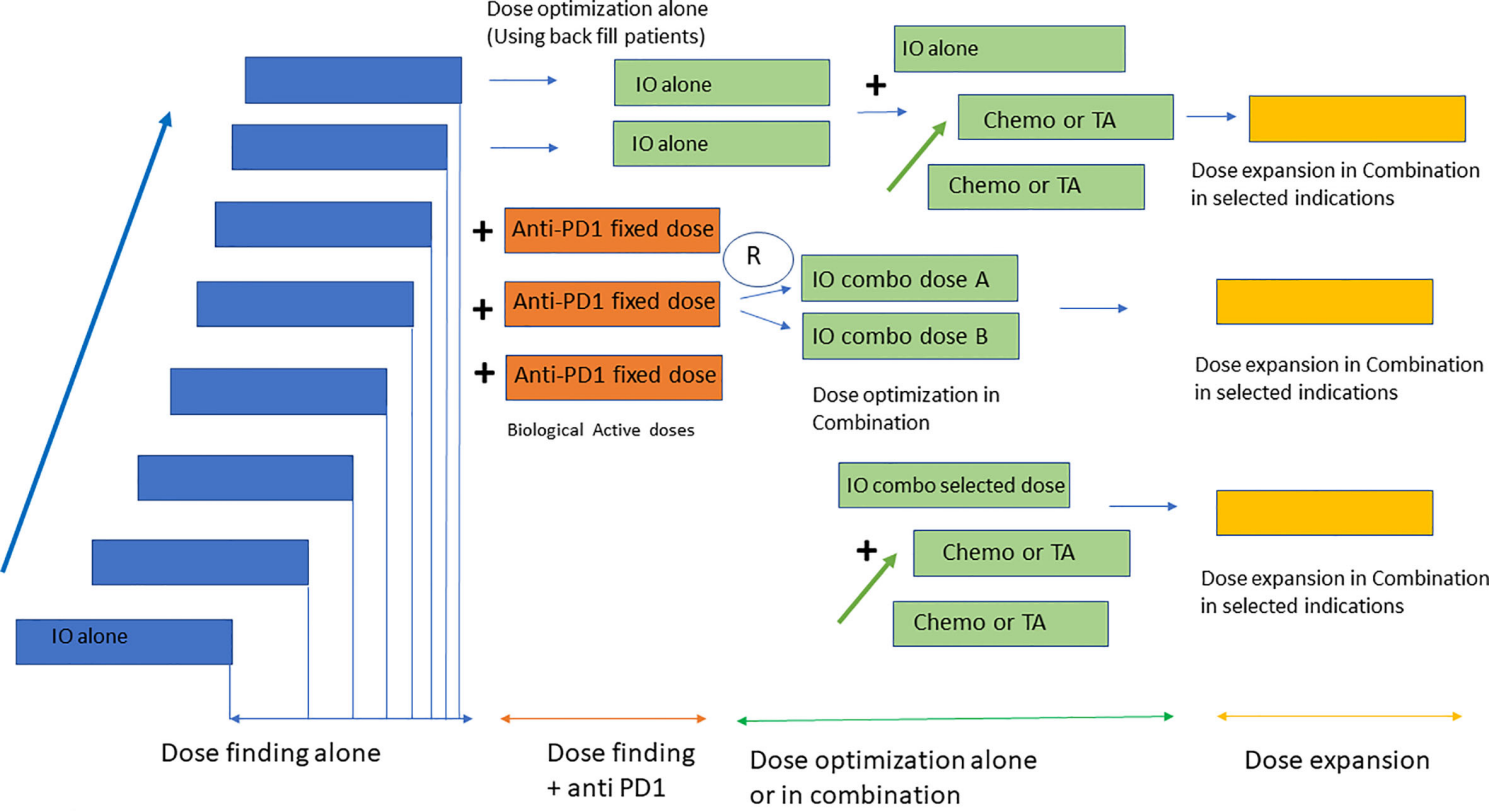
Design of early clinical studies with IO. Most phase I studies with IO consist of four parts including a dose escalation phase with the investigational agent alone, followed by a dose escalation in combination with anti PD1 at a fixed dose. Dose optimization strategies use back fill patients to identify the RP2D in monotherapy and randomization to two dose levels for the combination with anti PD1. Once the RP2D has been identified dose expansion cohorts in selected indications are conducted for probe of concept efficacy analysis.

In line with previous data, currently, most early-stage clinical studies particularly with IO use a master protocol approach for early development (45). This includes a single protocol in which several dose escalation parts alone or in combination, are followed by a multiple dose expansion, single-arm cohorts to identify early signs of activity (46, 47). This approach also aligns with several recent FDA requirements aiming at developing clinical strategies to better identify the dose selected for registration studies (48, 49). This initiative has been called the Optimus project (50). Of note, options for dose optimization vary depending on the mechanism of action of the compound, safety profile and combination strategies, and can include evaluation of different dose levels in the dose escalation phase using back-fill patients or selection of two different expansion cohorts with different dose levels. Dose optimization studies should be performed at biologically active doses where activity has been identified, and in indications with potential to detect clinical efficacy (48). The method for dose escalation is also relevant. Despite the availability of modern Bayesian designs for dose escalation, some studies still use the 3 + 3 design. This poses a significant problem for IO agents with stochastic toxicities that can appear in dose levels already previously thought to be safe and at times which can exceed the period of observation for dose limiting toxicity. Protocols with 3 + 3 design that do not take into account dose-limiting toxicities during the PK-PD expansion can result in challenges in dose selection. Bayesian Optimal Interval Design (BOIN) or modified toxicity probability interval (mTPI) are examples of dose escalation methods more appropriate for these studies (51, 52). **Figure 1** displays a summary of dose escalation phase I designs.

Finally, the specific pattern of response to IO agents must be taken in consideration particularly the expected changes in cross-sectional imaging. Modification to the response evaluation criteria in solid tumors to account for different response patterns in tumors than classic chemotherapy drugs (iRECIST) is of interest, but currently, these criteria are not considered by regulatory bodies for drug approvals (53).

## Endpoints for the development of IO agents

Randomized clinical trials with a time-to-event endpoint like overall survival (OS) or surrogates thereof such as progression free survival (PFS), have been the gold standard for the approval of novel anti-cancer agents (54, 55). More recently, in indications that constituted an unmet medical need or with low prevalence, single-arm phase 2 studies have been used to demonstrate clinical activity and support accelerated regulatory approval (54). Typically in these cases, the endpoint selected has been overall response rate (ORR) and/or median duration of response (mDoR), and only if benefit observed was considered as substantial especially in a particular clinical scenario where no active treatment was available, regulatory bodies have provided conditional approval (56, 57). Of note this approach is not specific to immunotherapy. Recently exceptional pathological complete responses (pCR) in specific tumor types have been considered as adequate for regulatory drug approval (58). However, in most of these situations a time to event endpoint such as PFS or OS was required for conversion to full approval, thereby requiring the completion of a phase III post-registration study comparing the new agent against the standard of care (SOC) (59). Examples are many in solid and hematologic malignancies, for instance the full approval of pembrolizumab in MSI-H colorectal cancer (60). In most cases, beneficial effect was confirmed in definitive phase 3 studies, but in some benefit could not be confirmed and this resulted in withdrawal of the approval of that agent for that indication (61). Examples of withdrawal include pembrolizumab (Keynote-604) (62) or nivolumab in extensive-stage small cell lung cancer (SCLC) (checkMate 451 and 331) that did not reach OS benefit in the phase III study (63, 64).

## Identification of a minimum magnitude of benefit in dose expansion cohorts: recent examples

In early clinical studies, once the biologically active dose has been identified, several expansion cohorts in specific tumor indications are initiated with the aim of finding signs of clinical activity. Expansion cohorts designed to explore for signals of activity should include a well-established population of patients powered with enough patients to detect activity. If an optimal study design is not followed, there is a risk that the potential benefit of the compound will be diluted as the most responsive population will not be included (65). It has been established that in a population of patients pretreated with anti-PD-(L)1 therapies, when rechallenging with anti-PD1 or other IO agent, response rates higher than 20% can be considered as meaningful, taken into consideration that single agent activity of anti PD-(L)1 antibodies produces responses in less than 10-15% of the patients (9). Therefore, it is generally accepted that a 20% ORR compared with SOC historical controls is the minimum necessary to consider the new agent with potential for further clinical development. Recently several examples have met this threshold. For instance, the anti-NKG2A antibody monalizumab demonstrated ORR of more than 20% in second line treatment in PD1-pretreated HNSCC patients in combination with cetuximab (66). Similarly, the ITL4 inhibitor MK4830 showed an ORR of more than 20% in PD1-pretreated patients in different solid tumors (67). Other examples include the anti-TIGIT antibody tiragolumab with significant activity in a specific expansion cohort of NSCLC patients (50% ORR and 80% disease control rate) (68) or the anti-LAG3 antibody relatlimab that demonstrated clinical activity in later treatment lines in melanoma before being explored in first-line (69). Very recently an anti-CTLA4 with an Fc enhanced fraction has demonstrated a very high rate of responses in tumors with relatively low immune-reactivity including ovarian cancer, sarcoma and microsatellite stable colorectal cancer (70). In this case, responses were higher than 30% in a heavily pretreated population where immunotherapy have never demonstrated clinical efficacy (70). In addition, activity has also been observed with anti-CD47 antibodies particularly in Myelodysplastic Syndrome (MDS) (71). An in-depth description of these studies is outside the scope of this review. However, in all these cases, the observed data support the further evaluation of these agents in more definitive trials.

## Late-stage clinical development

Once signs of clinical activity have been identified in early clinical studies, a late-stage clinical development plan is necessary. Either a randomized phase II study to confirm activity, or a phase II-III study with registration purposes can be designed. The anti-TIGIT antibody tiragolumab demonstrated significant clinical activity in a randomized phase II study in first-line PD-L1 positive NSCLC in combination with atezolizumab versus atezolizumab alone. The combination showed a median PFS of 5.4 months versus 3.6 months in the placebo plus atezolizumab group (72). These data support the development of a registration phase III study in first line NSCLC with two co-primary endpoints PFS and OS (73). Data for PFS and OS are expected to be released next year although the first interim analysis of PFS did not reach the defined threshold of activity (74). Similarly, negative results have been reported in combination with chemotherapy in first-line extensive stage small cell lung cancer (SCLC) (75). A different approach was taken for the development for the anti-LAG3 relatlimab where a combined phase II-III registration study was designed in first-line melanoma with a predefined futility analysis for activity in the phase II part (44).

Of note, some drugs have been explored in the early-stage/curable setting before demonstrating activity in metastatic/palliative patients. This can be due to strategic reasons from sponsors or may be guided by biological principles. In the field of small molecules only neratinib has received approval in the adjuvant setting before demonstration of benefit in the advanced stage (76). In the IO space, the anti-NKG2A monalizumab and the anti-CD73 oleclumab have been evaluated in locally advanced NSCLC in combination with durvalumab after chemoradiotherapy in stage III NSCLC (77). For both combinations an increase in ORR was observed compared with durvalumab alone after chemoradiotherapy (77) thereby supporting the current evaluation in larger phase III registration studies.

## Optimizing clinical development by patient selection and combinations

For a robust anti-tumor immune response, the existing patient immune system plays a central role, and modulation of the target outside tumor areas is key (78). This requisite has to be added to the presence of an immunoreactive tumor with high expression of the targets like PD-L1, TIGIT, or LAG3, as examples (9). For TCE therapies, recent data suggest the importance of the presence of pretreatment associated T-cell density with an important role of CD8+ T- cells and a negative implication of CD4+ T-cells or the presence of exhausted-like CD8+ T-cells (79–81).

Identification of biomarkers in liquid biopsy using circulating tumor DNA (ctDNA) has been used for stratification of risk and therefore potential response to anti-PD(L)1 therapies, like in locally advanced bladder cancer (82, 83). However, this is just an indirect measure of the tumor burden and not a direct evaluation of target engagement or correlates of the activated immune system. In line with this, inflammation is directly linked with a dysfunctional immune response (84). High pre-treatment levels of neutrophil to lymphocyte ratio (NLR) is an indirect measure of inflammation and can predict detrimental response to ICI (85, 86). Furthermore, the evaluation of the soluble form of PD-L1 in liquid biopsy has been implicated in detrimental response, but this finding was tumor dependent (87) and need further validation. In the future, it will be desirable to identify biomarkers of response but also biomarkers that could predict efficacy over time and that could be easily measured in plasma.

In line with this, given the fact that identification of a predictive biomarker is challenging, most agents under development are evaluated as a single agent or in combination with anti-PD(L)1 agents, in immune reactive tumors where anti-PD(L)1 agents are given alone in first line, including indications like PDL1+NSCLC or HNSCC tumors. Then, if activity is detected in single arm cohorts, expansion to other indications is explored. Description of novel combinations are beyond the scope of this work. However, it is important to mention those that act on exhausted T-cells as a principal cause of resistance, including 4-1BB or CD28 agonists (88, 89).

## Lessons learned

Given the lack of reliable animal models to predict efficacy in humans, decisions regarding the development of a particular agent, and the selection of indications to be explored, are usually based on the following criteria: i) the biological rationale of the target ii) the preclinical *in vitro* activity alone or in combination and iii) the presence of the target and the specific immune population in a particular tumor type. In case these criteria have been met for a particular agent, the potential for development of that compound will depend mainly on the mechanism of action and potential toxicity profile. Of note, toxicity will also depend on the mechanism of action. Substantial differences in toxicity have been observed with agents that modulate the myeloid compartment compared with those that activate T-cells. Toxicity of T-cell activating agents like T-cell engagers or bi-specific PDL1-41BB antibodies include severe infusion reactions or cytokine release syndrome, among others, rarely observed with the other type of agents (5). For an adequate trial design and an early clinical development plan, all these concepts must be taken in consideration including dose escalation, dose optimization and dose expansion strategies, in addition to the expected magnitude of benefit by indication.

In summary, the clinical development strategy for a particular compound should be designed from the early beginning, taken in consideration some of the topics that have been commented in this review.

## Author contributions

AO and AP have designed the study. AO, EA, and AT have contributed providing material. All authors contributed to the article and approved the submitted version.
